# Supplementary material for: Mass Spectrometry-Based Proteomic Profiling of a Silvaner White Wine
Source: Biomolecules. 2023 Apr 4;13(4):650. doi: 10.3390/biom13040650 (PMC10136162; doi:10.3390/biom13040650)
Supplement: Supplementary file 1 [file biomolecules-13-00650-s001.zip › Suplementary Figure1 and Tables S1 and S2.pdf]

Table S1. Comparison of proteins from the same Silvaner wine identified in the present study and proteins from a Silvaner wine identified by Albuquerque *et al.* [32].

**Proteins identified in both studies**	**Proteins identified only by Albuquerque et al. [32]**
**High-Abundance proteins**	**Low-Abundance proteins**
Class IV chitinase (27.5 kDa, *Vitis vinifera*)	Aspartyl protease family protein 1 (23.7 kDa, *Vitis vinifera*)
Glucan endo-1,3-β-glucosidase (23.9 kDa, *Vitis vinifera*)	Nucleoside diphosphate kinase (54.6 kDa, *Vitis vinifera*)
β-fructofuranosidase, soluble isoenzyme I (36.8 kDa, *Vitis vinifera*)	Putative glutathione S-transferase (11.7 kDa, *Vitis vinifera*)
Non-specific lipid-transfer protein (63.5 kDa, *Vitis vinifera*)	Aquaporin PIP2-2 (37.7 kDa, *Vitis vinifera*)
Thaumatin-like protein (32 kDa, *Vitis vinifera*)	Barwin domain-containing protein (27.3 kDa, *Vitis vinifera*)
Endochitinase EP3 (26 kDa, *Vitis vinifera*)	Fructose-bisphosphate aldolase (14.1 kDa, *Vitis vinifera*)
**Low-Abundance proteins**	Tetraspanin-8 (82.4 kDa, *Vitis vinifera*)
Plasma membrane ATPase (13.4 kDa, *Vitis vinifera*)	Protein kinase domain-containing protein (60.7 kDa, *Vitis vinifera*)
Ubiquitin-60S ribosomal protein L40 (79.3 kDa, *Vitis vinifera*)	Profilin-1 (41.9 kDa, *Vitis vinifera*)
LysM domain-containing GPI-anchored protein 1 (34.4 kDa, *Vitis vinifera*)	Early nodulin-like protein 2 (35.7 kDa, *Vitis vinifera*)
	11S globulin seed storage protein 2 (33.7 kDa, *Vitis vinifera*)
	Putative inactive receptor kinase (52 kDa, *Vitis vinifera*)
	Tonoplastic transporter 1 (16.9 kDa, *Vitis vinifera*)
	Pectinesterase/pectinesterase inhibitor 3 (42.4 kDa, *Vitis vinifera*)
	Cysteine proteinase RD21A (30.7 kDa, *Vitis vinifera*)
	LysM domain-containing GPI-anchored protein 2 (105.9 kDa, *Vitis vinifera*)
	Glycerophosphodiester phosphodiesterase GDPDL4 (69.5 kDa, *Vitis vinifera*)
	Integrase catalytic domain-containing protein (112.2 kDa, *Vitis vinifera*)
	X8 domain-containing protein (61.6 kDa, *Vitis vinifera*)
	PINc domain-containing protein (50.9 kDa, *Vitis vinifera*)
	Glyceraldehyde-3-phosphate dehydrogenase (104.7 kDa, *Vitis vinifera*)
	Peroxidase (37.4 kDa, *Vitis vinifera*)
	PI-PLC X domain-containing protein (80.1 kDa, *Vitis vinifera*)
	Fasciclin-like arabinogalactan protein 10 (43.7 kDa, *Vitis vinifera*)
	FAS1 domain-containing protein (16.3 kDa, *Vitis vinifera*)
	PMEI domain-containing protein (38.6 kDa, *Vitis vinifera*)
	Heat shock cognate 70 kDa protein (73.5 kDa, *Vitis vinifera*)
	Expansin-like EG45 domain-containing protein (23.6 kDa, *Vitis vinifera*)

Table S2. Comparison of proteins from different white wines reported in the literature and identified in the present study. Bold marked proteins are found in all studies.

Semillon	German Portugieser	Chardonnay	Sauvignon Blanc
**Marangon *et al.*** [38]	**Wigand *et al.*** [15]	**Cilindre *et al.*** [22]	**Kwon** [30]
			Acid phosphatase (*Saccharomyces cerevisiae*)
**Class IV endochitinase (*Vitis vinifera*)**	**Class IV endochitinase (*Vitis vinifera*)**	**Class IV endochitinase (*Vitis vinifera*)**	**Class IV endochitinase (*Vitis vinifera*)**
		Endo-(1,3)-*β*-glucanase (*Vitis vinifera*)	Endo-(1,3)-*β*-glucanase (*Vitis vinifera*)
	Glycosidase (*Saccharomyces cerevisiae*)		Glycosidase (*Saccharomyces cerevisiae*)
			Target of Sbf (*Saccharomyces cerevisiae*)
**Thaumatin-like Protein (*Vitis vinifera*)**	**Thaumatin-like Protein (*Vitis vinifera*)**	**Thaumatin-like Protein (*Vitis vinifera*)**	**Thaumatin-like Protein (*Vitis vinifera*)**
**Vacuolar invertase (*Vitis vinifera*)**	**Vacuolar invertase (*Vitis vinifera*)**	**Vacuolar invertase (*Vitis vinifera*)**	**Vacuolar invertase (*Vitis vinifera*)**
	Cell wall protein (*Saccharomyces cerevisiae*)		
	Lipid transfer protein (*Vitis vinifera*)		
			Daughter-specific cell protein (*Saccharomyces cerevisiae*)

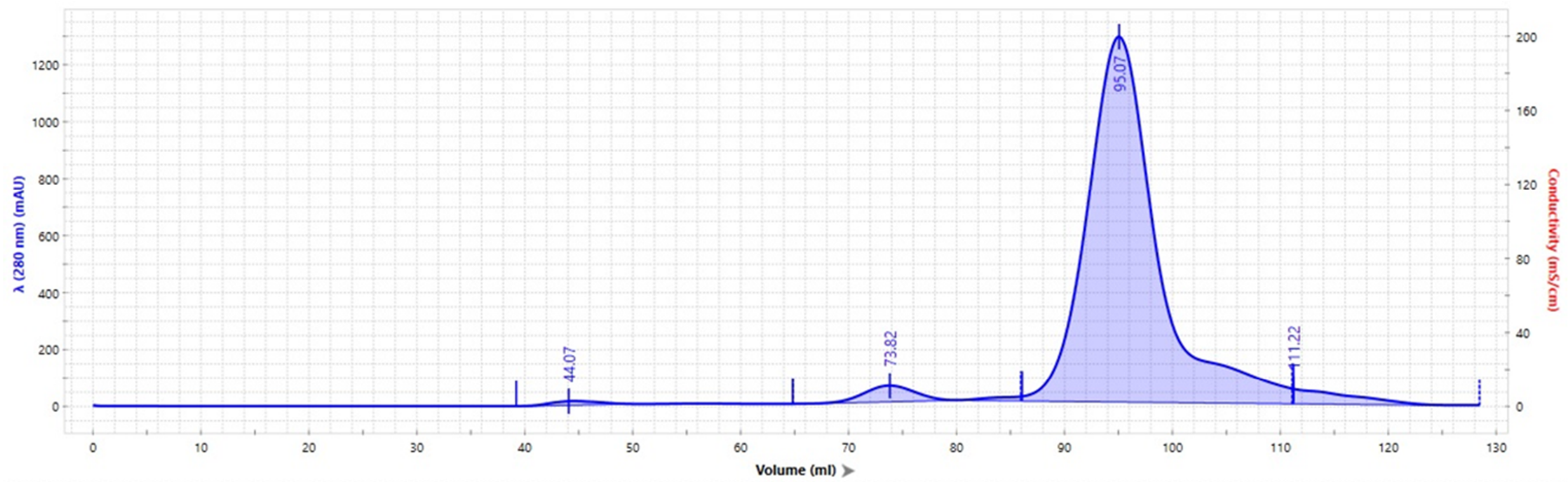

Runs/Traces | Peaks | Attachments

Peak Number	Start (ml)	End (ml)	Retention Time (min)	Retention Volume (ml)	Height (mAU)	Area (ml*mAU)	Relative Area (%)	Width at Half Height (ml)	Peak Asymmetry	Fractions	Peak Type	Path Length (cm)	Molecular Mass (kDa)	Extinction Coefficient ((mg/ml)$^{-1}$ cm^{-1})
Run: SEC 2 WP for proteomics, Type: λ (280 nm)														
1	39.21	64.82	48.97	44.07	14.79	85.88	0.83	6.04	1.34		Manual	0.5		
2	64.82	85.97	82.01	73.82	57.55	366.80	3.53	5.27	2.62		Manual	0.5		
3	86.08	111.10	105.61	95.07	1283.01	9615.41	92.50	6.11	1.71		Manual	0.5		
4	111.22	128.45	123.54	111.22	52.43	327.22	3.15	5.48	0.00		Manual	0.5		

Protein initial concentration : 0.5±0.1 mg/mL

Figure S1. Quantification of the % of yield from the sample to protein fraction after FPLC fractionation based on the relative area calculated by the software ChromLab v6.1.29).
